# Genome-Wide Identification of the *LHC* Gene Family in Kiwifruit and Regulatory Role of *AcLhcb3.1/3.2* for Chlorophyll a Content

**DOI:** 10.3390/ijms23126528

**Published:** 2022-06-10

**Authors:** Juan Luo, Muhammad Abid, Jing Tu, Puxing Gao, Zupeng Wang, Hongwen Huang

**Affiliations:** 1 College of Life Science, Nanchang University, Nanchang 330031, China; luojuan0118@163.com (J.L.); tujing0819@163.com (J.T.); 2 Lushan Botanical Garden, Chinese Academy of Sciences, Jiujiang 332900, China; muhammadabid@lsbg.cn (M.A.); gaopuxin_3290@163.com (P.G.); 3 Engineering Laboratory for Kiwifruit Industrial Technology, Chinese Academy of Sciences, Wuhan 430074, China

**Keywords:** *Actinidia chinensis*, *Actinidia eriantha*, light-harvesting chlorophyll a/b-binding protein, expression profiles, *AcLhcb3.1/3.2*, chlorophyll content

## Abstract

Light-harvesting chlorophyll a/b-binding (LHC) protein is a superfamily that plays a vital role in photosynthesis. However, the reported knowledge of LHCs in kiwifruit is inadequate and poorly understood. In this study, we identified 42 and 45 *LHC* genes in *Actinidia chinensis* (Ac) and *A. eriantha* (Ae) genomes. Phylogenetic analysis showed that the kiwifruit LHCs of both species were grouped into four subfamilies (Lhc, Lil, PsbS, and FCII). Expression profiles and qRT-PCR results revealed expression levels of *LHC* genes closely related to the light, temperature fluctuations, color changes during fruit ripening, and kiwifruit responses to *Pseudomonas syringae* pv. *actinidiae* (Psa). Subcellular localization analysis showed that AcLhcb1.5/3.1/3.2 were localized in the chloroplast while transient overexpression of AcLhcb3.1/3.2 in tobacco leaves confirmed a significantly increased content of chlorophyll a. Our findings provide evidence of the characters and evolution patterns of kiwifruit *LHCs* genes in kiwifruit and verify the *AcLhcb3.1/3.2* genes controlling the chlorophyll a content.

## 1. Introduction

Green plants convert light energy into chemical energy required to carry out cellular processes through photosynthesis [1]. Chlorophyll participates in the photosynthesis process by capturing and transferring light energy [2]. Light-harvesting chlorophyll a/b-binding (LHC) proteins play a role in capturing light during photosynthesis, micro-organization and photoprotection of photosystem II (PSII), and alleviation of oxidative stress [2,3,4]. The LHC superfamily is a plant-specific superfamily that comprises four subfamilies, namely light-harvesting chlorophyll a/b-binding protein (Lhc), light-harvesting-like (Lil), photosystem II subunit S (PsbS), and ferrochelatase II (FCII) [5]. The Lhc subfamily is further divided into two groups, Lhca and Lhcb. Similarly, the Lil subfamily consists of four distinct groups, including one-helix protein (OHP), stress-enhanced protein (SEP), early light-induced protein (ELIP), and photosystem II protein 33 (Psb33). However, PsbS and FCII subfamily include only a single group [6]. The chlorophyll a/b binding domain (PF00504) is widely found in *LHC* superfamily members in plant species [7]. So far, *LHC* superfamily members have been identified in different plant species, including *Arabidopsis*, *Oryza sativa*, *Carica papaya*, *Gossypium hirsutum*, *Manihot esculenta*, and *Ricinus communis* [7,8,9,10]. 

Structural and functional analyses of *LHC* superfamily members showed their involvement in light-harvesting and response to various stresses. The overexpression of tomato *LeLhcb2* in tobacco revealed an elevated tolerance to chilling stress and the alleviated photo-oxidation of PSII [11]. Enhanced expressions of tomato *LHC* genes in RNAi-*SlBEL11* tomato fruits were related to increased chlorophyll contents [12], while overexpression of *Sedum alfredii* *SaLhcb2* in tobacco increased the shoot biomass and higher Cd^2+^ accumulation [13]. In *Arabidopsis thaliana*, *AtLhcb* members regulated ABA (abscisic acid) induced stomatal movement [14], seed germination and post-germination growth, and plant adaptation to environmental changes [15,16]. Additionally, *AtLhcb1* and *AtLhcb2* exhibited different but complementary functions during phosphorylation-driven state transitions in photosynthetic light-harvesting in *Arabidopsis* [17]. In *Apium graveolens*, up-regulation expression of the *AgLhcb1* gene enhanced its photosynthetic efficiency, suggesting that this gene could be used as the marker for estimating the photosynthetic rate [18]. In rice, iron deficiency significantly depressed expressions of *OsLhca1/2/3/4* and caused a great decrease in chlorophyll content and photosynthetic efficiency [19]. Carbon dioxide treatment in cucumber also increased photosynthetic efficiency by enhancing *LHC* gene expression levels [20]. 

In green plants, the high chlorophyll contents and a robust stay-green trait can improve the photosynthetic efficiency of plants [21]. The stay-green phenotype, which breeders have targeted, relies on the expression of *LHCs* and chlorophyll content [22]. The chlorophyll content of the early senescence leaf (*esl*) mutant in rice significantly decreased, revealing extremely depressed expressions of *LHC* superfamily genes [23]. The overexpression of the tea *CsLhc* gene in *Arabidopsis Lhcb* mutant facilitated chlorophyll accumulation and promoted leaf regreen by increasing expression levels of chlorophyll biosynthesis-relative genes [24]. Similarly, the overexpression of the apple *MdLhcb4.3* gene also enhanced chlorophyll contents in *Arabidopsis* [25]. The knockout mutant plants of *AtLhcb6* and *AtLhcb5*, and *AtLhcb4* exhibited significantly lower chlorophyll contents in *Arabidopsis* [3,26].

Kiwifruit is one of the most successfully domesticated fruit trees of the 20th century and has become a popular fruit with high nutritional value [27]. Interestingly, kiwifruit variations in fresh colors were caused by the contents and proportions of chlorophylls, carotenoids, and anthocyanins, which regulate green, yellow, and red flesh [28]. Expression levels of *LHC* genes were previously found to be positively correlated to chlorophyll content during kiwifruit ripening, suggesting that *LHC* genes may regulate the stay-green flesh of ripening fruits [29]. However, the identification and characterization of kiwifruit *LHC* genes have not been reported, and the roles of kiwifruit *LHC* genes in regulating chlorophyll content need to be investigated. We report a genome-wide identification of the *LHC* superfamily members in *Actinidia chinensis* cv. Red5 (Ac, red flesh fruit due to chlorophyll degradation during fruit ripening) and *A. eriantha* cv. White (Ae, green flesh fruit maintaining chlorophyll during fruit ripening). We performed gene structures, evolutionary relationships, protein motifs, and gene expression profile analysis in different kiwifruit tissues and different stress treatments to elucidate the structural and functional evolution of kiwifruit *LHCs*. Moreover, we performed transient overexpression of *AcLhcb1.5/3.1/3.2* genes in *Nicotiana benthamiana* to investigate the potential roles of *AcLhcb1.5/3.1/3.2* in regulating chlorophyll content. Our study provides valuable information for kiwifruit *LHC* superfamily genes and defines the potential roles of *AcLhcb1.5/3.1/3.2* in modulating chlorophyll metabolism. Additionally, we have identified potential candidate *LHC* genes that can provide a valuable source for improving chlorophyll contents and maintaining photosynthesis in plants under stress conditions.

## 2. Results

### 2.1. Genome-Wide Identification and Phylogenetic Analysis of Kiwifruit LHCs 

We firstly retrieved sequences of AtLHC proteins, and those sequences were further used as a query to search Ac and Ae proteins using the Blastp tool [26]. Proteins identified in Ac and Ae were confirmed by the conserved domain analysis, and we identified 42 and 45 putative LHCs from Ac and Ae genomes, respectively (Table S2). The kiwifruit LHC proteins were named after AtLHC proteins. 

**Figure 1:**
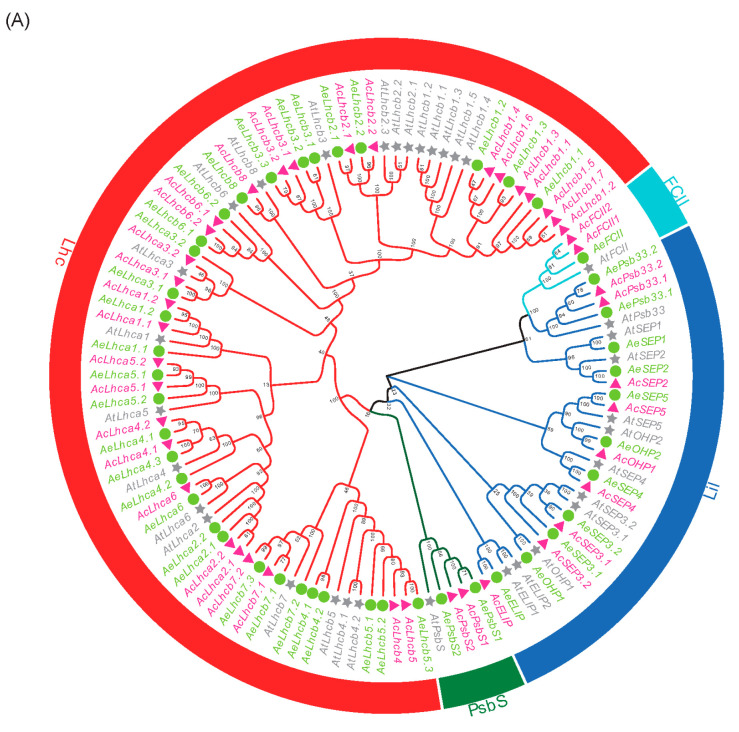

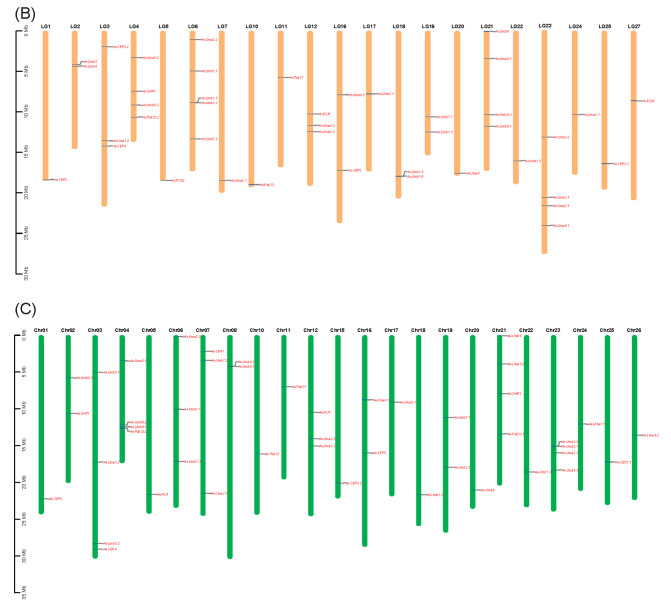
Phylogenetic tree of LHC proteins (**A**) and distribution of *LHC* genes in Ac (**B**) and Ae (**C**) chromosomes. The full-length LHC protein sequences from *Arabidopsis* (*At*, gray gene name and pentagram), *A. chinensis* (Ac, deep pink gene name and triangle), and *A. eriantha* (Ae, lawn green, and circle) were aligned using ClustalX 2.0 with default parameters. Then, the unrooted phylogenetic tree was constructed using MEGA X and the Neighbour-Joining method. The Lhc subfamily, PsbS subfamily, Lil subfamily, and FCII subfamily were highlighted using red, green, royal blue, and aqua sectors.

Phylogenetic analyses were performed for 42 AcLHCs, 45 AeLHCs, and 34 AtLHCs proteins to explore the phylogenetic relationship and evolutionary pattern of kiwifruit *LHC* genes. Consistent with the classification of AtLHC proteins, kiwifruit LHC proteins were grouped into four distinct subfamilies, namely Lhc, Lil, PsbS, and FCII (Figure 1A). Our results show that the Lhc subfamily had the most members while FCII had the least (Figure S1). In the kiwifruit genome, the *AcLHC* genes were randomly distributed on 21 chromosomes (Figure 1B), of which chromosome 6 included the maximum gene number (five *AcLHC* genes), while gene numbers varied between 1 and 3 for the rest of the chromosomes (Figure S2A). Similarly, the *AeLHC* genes were unevenly distributed on 23 chromosomes (Figure 1C), and gene numbers varied between 1 and 4 for all chromosomes (Figure S2B).

### 2.2. Multiple Sequence Alignment and Analysis of Kiwifruit LHCs Structure 

**Figure 2:**
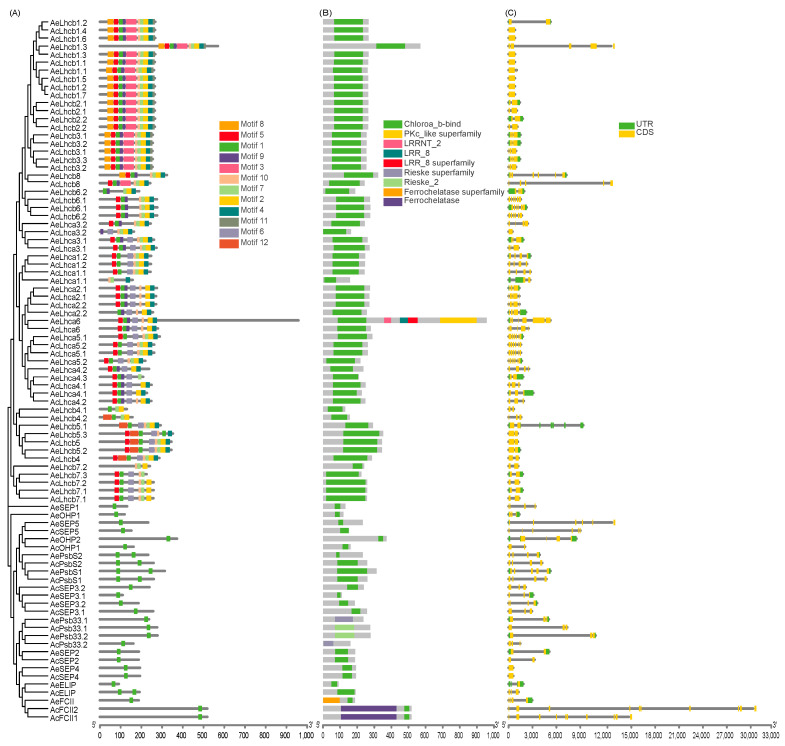
Gene structure and conserved motif architecture of LHC family in two kiwifruit species. (**A**) motifs contained in AcLHC and AeLHC proteins; (**B**) showed conserved domain distribution; (**C**) the exon-intron structure of kiwifruit *LHC* genes.

The conserved motifs among kiwifruit LHCs were identified to infer structural variation and possible functional divergence. We identified 12 conserved motifs (designated as motifs 1–12) in kiwifruit LHCs (Figure 2A and Figure S3). The results show that kiwifruit LHC proteins with closer phylogenetic relationships had more similar motif architectures (Figure 2A). The Lhc subfamily contained the highest motif number compared to other subfamilies (Figure S4). Motif 1 was presented in all kiwifruit LHC proteins (Figure 2A) and located in the conserved Chloroa_b-bind domain (Figure 2B). 

Intron numbers of kiwifruit *LHCs* varied from zero to nine, while corresponding exon numbers ranged from one to ten (Figure 2C). In addition, exon-intron structures and gene length of kiwifruit *LHCs* varied, especially for genes belonging to Lil, PsbS, and FCII subfamilies, indicating that gene structures might drive gene function divergence of kiwifruit *LHCs* (Figure 2C). The comparison of exon-intron structure in genes could provide insights into evolutionary mechanisms underlying the formation of gene families in plants [30]. 

### 2.3. Synteny Analysis and cis-Element of Kiwifruit LHCs

The collinearity analysis was carried out to visualize the synteny relationships among homologous *LHCs* and infer gene duplication events. Eighteen duplicated gene pairs were identified in Ac and Ae (Figure S5). The Ka/Ks ratio for duplicated gene pairs varied from 0.02 to 0.47, with an average of 0.18 (Table S3), suggesting that all duplicated gene pairs underwent purifying selection. In addition, the Ks value of duplicated gene pairs varied from 0.10 to 1.37 (Table S3), suggesting that duplicated pairs have different evolution rates. The higher Ks value of dispersed duplication than whole-genome duplication (WGD) suggested that it may have happened earlier than the WGD event. Most duplicated gene pairs underwent WGD, indicating that WGD played a vital role in expanding *LHCs* in kiwifruit. 

The potential cis-regulatory elements were predicted in the 2.0 kb upstream sequence of *AcLHCs* and *AeLHCs*. In total, 509 and 500 cis-elements were identified in promoter regions of *AcLHCs* and *AeLHCs* and further classified into nine types, including light-responsive element, auxin-responsive element, wound-responsive element, defense- and stress-responsive element, abscisic acid-responsive element, MeJA-responsive element, low-temperature-responsive element, gibberellin-responsive element, and salicylic acid-responsive element (Figure S6). Light-responsive cis-element and low temperature-responsive cis-element were the most abundant in both kiwifruit species, indicating that light and low temperature could significantly regulate *LHCs* expression patterns (Figure S6). 

### 2.4. AcLHC Genes Regulated Kiwifruit Responses to Biotic and Abiotic Stresses

**Figure 3:**
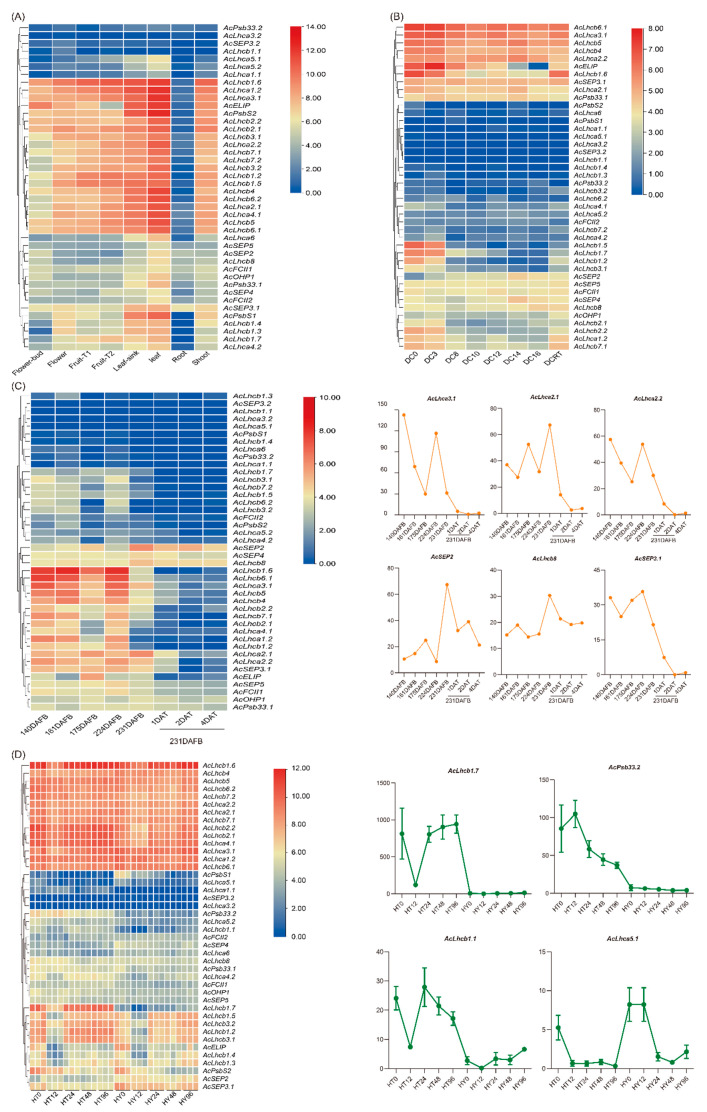
Expression profiles of *AcLHC* genes in different tissues, fruit developmental stages, and under different treatments. The bar at the right of each heat map represents expression values. (**A**) Expression profiles of *AcLHCs* in eight different tissues (flower bud, flower, fruit T1: no ethylene production, fruit T2: autocatalytic ethylene production, leaf sink, leaf, root, and shoot). (**B**) Expression profiles of *AcLHCs* in mature fruit exposed to eight different temperatures in storage for two days. DC, degrees celsius; RT, room temperature. (**C**) Expression profiles of *AcLHCs* in the fruit developmental stages and samples treated with ethylene. DAFB, days after the full bloom of fruit; DAT, day after being treated with ethylene. (**D**) Expression profiles of *AcLHCs* in two kiwifruit cultivars infected with Psa. Huate (HT) and Hongyang (HY) represented resistant and susceptible cultivars, respectively. The number following the cultivar’s name showed hours post the Psa invasion.

We used four different transcriptome datasets to assess the expression patterns of *AcLHCs*. To estimate the expression bias of *AcLHCs* in different kiwifruit tissues, we analyzed 42 genes in eight different tissues (flower bud, flower, fruit T1: no ethylene production, fruit T2: autocatalytic ethylene production, leaf sink, leaf, root, and shoot) (Figure 3A). Our results suggest that *AcLHCs* exhibited tissue-specific expression patterns (Figure 3A). Interestingly, *AcLHCs* were abundantly expressed in green tissues (such as leaves) compared to non-green tissues (such as roots) (Figure 3A). Low temperature affects the plant chlorophyll content [28], and we explored the effect of low temperature on *AcLHCs* expression levels (Figure 3B). The results reveal that *AcLHC* members respond to low-temperature regimes differently (Figure 3B). We identified three candidate genes (*AcLhcb1.5*, *AcLhcb1.7*, and *AcLhcb1.2*) as significantly responsive to low temperatures, and low-temperature responsive elements were identified in promoter regions of those three genes (Figure 3B and Figure S6). 

To infer the potential functions of *AcLHCs* in regulating kiwifruit development and response to ethylene treatment, we re-analyzed the expression profiles of *AcLHCs* in different fruit developmental stages treated with or without ethylene (Figure 3C). The results reveal that the expression levels of five *AcLHCs* (*AcLhca3.1/2.1/2.2*, *AcSEP2*, and *AcSEP3.1*) were significantly down-regulated and one *AcLHC* (*AcLhcb8*) was down-regulated with the ethylene treatment (Table S4), verifying that those six *AcLHCs* could respond for kiwifruit degreening caused by the ethylene treatment [31]. 

A previous study suggested that chlorophyll content significantly affected plant resistance to bacterial pathogens [32]. We assessed expression profiles for *AcLHCs* after Pseudomonas syringae pv. actinidiae (Psa) inoculation in a resistant cultivar (Huate, HT) and a susceptible cultivar (Hongyang, HY), revealing expression levels of three genes (*AcLhcb1.7*, *AcPsb33.2*, and *AcLhcb1.1*) significantly up-regulated, but one gene (*AcLhca5.1*) significantly down-regulated in HT compared to HY. Our results suggest that these four genes played an essential role in the resistance of kiwifruit to Psa (Figure 3D). It will be interesting to investigate genome environment associations (GEAs) to identify adaptive variations between green and non-green flesh kiwifruit [33,34,35].

### 2.5. RT-qPCR Validation of Kiwifruit LHCs in Different Tissues

**Figure 4:**
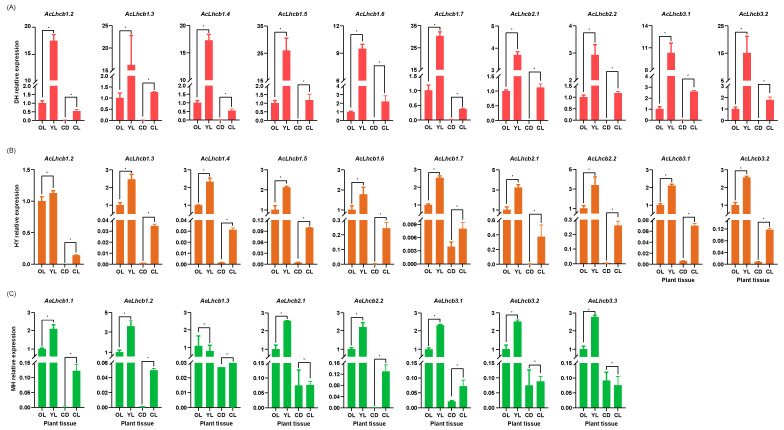
Expression analysis of *AcLHC* and *AeLHC* genes using RT-qPCR in different tissues. Actin was used as the internal standard for each gene. (**A**) for DH, (**B**) for HY, and (**C**) for MH. The results are shown as mean values and standard deviation of three biological replicates of different tissues and three technical replicates in each biological sample. The *y*-axis was the relative expression level. * indicated significant differences accorded to Tukey’s multiple range tests (*, *p* < 0.05). OL, old leaves; YL, young leaves; CD, callus tissues under dark condition; CL, callus tissues under light condition; DH, ‘Donghong’; HY, ‘Hongyang’; MH, ‘Maohua no.1’.

We selected ten *AcLHCs* and eight *AeLHCs* to perform RT-qPCR analysis in old and young leaves (OL, YL) and callus under light and dark conditions (CL, CD) (Figure S7). Plant samples used for this analysis were collected from Ac cultivars ‘Donghong’ (DH) and ‘Hongyang’ (HY) and Ae cultivar ‘Maohua no.1’ (MH) (Figure S7). Our results show that all the selected kiwifruit *LHC* genes were more expressed in leaves than calluses. The gene expressions were relatively higher in young leaves than in old leaves, except *AeLhcb1.3* in MH, suggesting that those genes were primarily expressed in green tissues, consistent with the first transcriptome results (Figure 3A and Figure 4). Additionally, expression levels of all selected *AcLHC* genes in calluses under light conditions were higher than those in calluses under dark, suggesting that light induces higher expression of those *AcLHC genes*, which is confirmed by the identification of the light-responsive elements in promoter regions (Figure 4A,B and Figure S6). Four out of eight *AeLHC* genes were also induced by the light in calluses (Figure 4C). Interestingly, the relative expression levels of eight *AcLHC* genes (*AcLhcb1.2*/*1.3*/*1.4*/*1.5*/*1.6*/*1.7*/*3.1*/*3.2*) in DH were higher than those in HY, revealing significant differences among different cultivars (Figure 4A,B).

### 2.6. Subcellular Localization of Kiwifruit LHC and Transient Transformation of AcLhcb Genes in Tobacco Leaves

**Figure 5:**
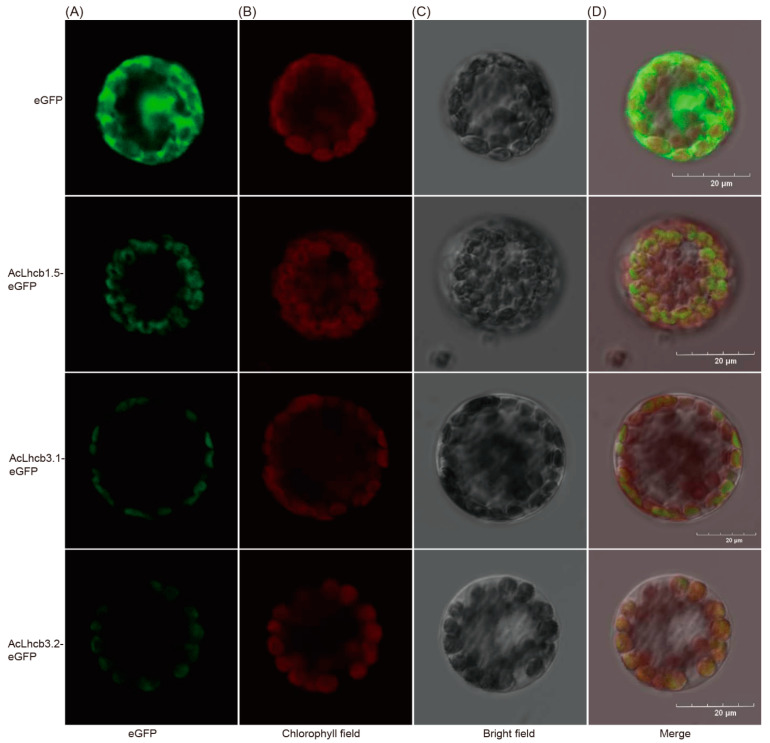
Subcellular localization of the fusion protein 35S::AcLhcb1.5/3.1/3.2::eGFP in mesophyll protoplasts of *Arabidopsis*. Images were taken under (**A**) fluorescence, (**B**) chlorophyll field, and (**C**) bright field. (**D**) Merged images (**A**–**C**) of representative cells expressing eGFP. The chlorophyll autofluorescence was used to localize chloroplasts. Bars = 20 μm.

**Figure 6:**
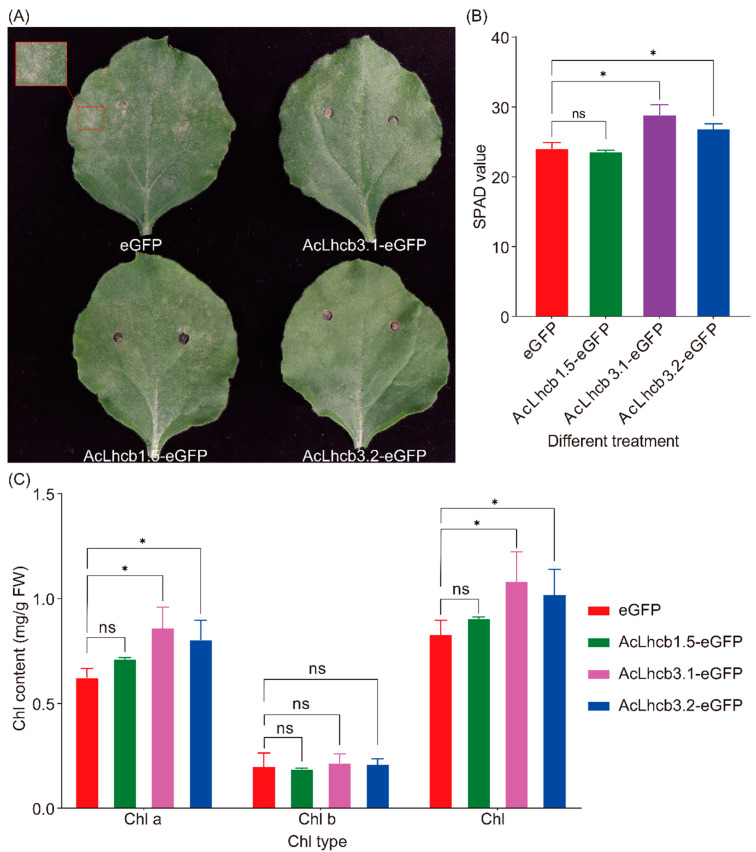
Transient expression of AcLhcb1.5/3.1/3.2 in tobacco leaves. (**A**) Growth phenotypes of 6-weeks-old soil-grown tobacco leaves of control (eGFP) and LHC overexpression (AcLhcb1.5-eGFP, AcLhcb3.1-eGFP, and AcLhcb3.2-eGFP) for 4 d. (**B**) Each treatment’s SPAD value and (**C**) chlorophyll content was measured on the fourth day after transformation. The results are shown as means and SDs from three independent experiments. For (**B**,**C**) * indicate significant differences between treatments, ns indicate no significance, according to two-way ANOVA and Tukey’s multiple range tests (*, *p* < 0.05).

We selected three candidate genes (*AcLhcb1.5/3.1/3.2*) based on the difference in significance levels of mean differences for RT-qPCR data from leaves (OL and YL) and calluses (CL and CD). In-silico analysis for subcellular localization of AcLhcb1.5/3.1/3.2 predicted their presence in the chloroplast. Then, we further confirmed the localization of candidate gene products by performing transient transformation of *Arabidopsis* leaf protoplasts and tobacco leaves (Figure 5and Figure S8). We performed transient expression of *AcLhcb1.5/3.1/3.2* in tobacco leaves to assess their potential role in chlorophyll content. Our results show that tobacco leaves which transiently expressed *AcLhcb3.1/3.2* had significantly higher SPAD value (Soil and Plant Analyzer Development), chlorophyll a content, and total chlorophyll content. Still, no significant change was found in the content of chlorophyll b. This result is consistent with *GhLhcb2.3* influence on the synthesis of chlorophyll a [9]. In contrast, *AcLhcb1.5* did not affect chlorophyll content in tobacco leaves (Figure 6). The tobacco leaves transiently expressed *AcLhcb1.5/3.1/3.2* had a higher value of Chl a/b than control (Figure S9).

## 3. Discussion

During photosynthesis, the chlorophyll-LHCs complexes located at PSI and PSII capture light to convert light energy into chemical energy required for various cellular processes in plants [36]. However, free chlorophylls and their metabolic intermediates can produce harmful reactive oxygen species even under normal conditions [2]. The dynamic balance of chlorophylls is closely related to the synthesis, degradation, maintenance of chlorophylls, and the dismantling and assembly of the PSI and PSII complexes [37]. A previous report suggested that the application of CO_2_ improved the chlorophyll content by upregulating chlorophyll-related genes, particularly *LHCs*, in cucumber plants [20]. The LHC family members have been identified in different plant species, i.e., 34 in *Arabidopsis*, 29 in *Oryza sativa*, 28 in *Carica papaya*, 55 in *Gossypium hirsutum*, 35 in *Manihot esculenta*, and 28 in *Ricinus communis* [5,7,8,9,10]. In the present study, we performed the genome-wide analysis of *LHC* gene family members in two different diploid kiwifruit species (Ac and Ae) and identified 42 *LHC* genes in Ac and 45 *LHC* genes in Ae (Table S1). Thus, *Actinidia* species have more *LHCs* than most reported plants. Interestingly, none of the kiwifruit *LHCs* genes was clustered with *AtSEP1* (Figure 1). We speculate that the kiwifruit genome underwent loss of the *SEP1* gene during the kiwifruit *LHC* family evolution process.

The WGD events were responsible for the expansion of kiwifruit *LHC* family members except for *AcLhcb1.1*, which could experience dispersed duplication events (Figure S5 and Table S3). Our finding confirms that both Ac and Ae had experienced three ancient WGD events [38]. The difference in the numbers of *LHCs* between *A. thaliana* or other plants and kiwifruit is probably due to the number of WGD events (two in *A. thaliana* and three in kiwifruit). However, the difference in the number of *LHC* members in the superfamily and subfamilies of Ac and Ae suggests that they might have experienced different evolution patterns. In addition, the Ka/Ks ratios of the 18 duplicated pairs in both *AeLHCs* and *AcLHCs* revealed that *LHC* family members had experienced purifying selection during the evolution process of both species. 

The chlorophyll a/b-binding domain is highly conserved in the LHC family in different plants [5]. In the present study, all kiwifruit LHC family members have chlorophyll a/b-binding domain except AcPsb33.1, AcPsb33.2, AePsb33.1, and AePsb33.2, which possessed the Rieske domain similarly to AtPsb33 (Figure 2). It would be interesting to know if LHCs without the chlorophyll a/b-binding domain play a role identical to LHCs containing this domain. Kiwifruit *LHC* genes in the Lhc subfamily contained similar motifs and intron-exon structures, indicating that this subfamily had a highly conserved structural evolution (Figure 2). However, this phenomenon was not found in the other three subfamilies of LHC.

Our analysis of the promoter region of kiwifruit *LHC* genes found that kiwifruit *LHCs* differ greatly in the composition of cis-acting elements even within the same subfamily (Figure S6). The cis-element arrangements of the orthologous *LHC* gene pairs for both species of kiwifruit displayed a marked difference, suggesting that the orthologous *LHC* gene pairs possibly had different response mechanisms for biotic and abiotic stress in kiwifruit plants. In addition, the type and number of *cis*-element significantly varied in *AcLHCs* and *AeLHCs*, indicating that *LHCs* could function independently or synergistically to promote the normal growth of plants under different environmental conditions. It is reasonably speculated that within the same subfamily of *LHC* genes, expression was regulated through different transcriptional expression patterns, and our transcriptome analysis results are consistent with this speculation (Figure 3). All kiwifruit *LHC* genes had multiple light-responsive elements, suggesting a similar photoprotection function of the HvLhcb1 protein [39]. Additionally, we found a variety of hormone and stress-related elements in the promoter region of kiwifruit *LHC* genes. Previously, it has been reported that the expression of *LHC* genes was related to the hormone and abiotic stress. For instance, high salinity and low temperature reduced the expression of most *ZmLhca* and *ZmLhcb* genes [40]. Low concentration of ABA promoted *AtLhcb1~6* expression, but high concentration inhibited *AtLhcb1~6* expression [41]. The *AtLhcb1~6* mutant had a poorer tolerance to drought and showed lower reactive oxygen species (ROS) after ABA treatment than the wild type [14]. Thus, we believe that cis-acting elements in the promoter region could be responsible for regulating kiwifruit *LHCs* expression.

Characterization of gene expressions in different plant organs under different conditions can be valuable for identifying candidate genes for desired breeding traits. In the present study, the kiwifruit *LHC* genes exhibited low expression in CL and almost no expression in CD, similar to low expression of *LHC* genes in *esl* mutant rice compared to wildtype [23]. Moreover, significant differences in kiwifruit *LHC* gene expression profiles, such as AcLhcb1.5/1.7, for different cultivars suggested that gene expression was influenced by the different cultivars’ genetic backgrounds.

The subcellular localization analysis of AcLhcb1.5/3.1/3.2 proteins in tobacco and *Arabidopsis* confirmed their presence in chloroplasts (Figure 5 and Figure S8), supporting the argument that these three proteins might be involved in photosynthesis. As expected, the transformation of AcLhcb3.1/3.2 proteins in tobacco leaves enhanced the SPAD value and chlorophyll a content. 

It is essential to know the molecular regulation mechanism of crops to cope with the adverse effects of climate change on food safety and agricultural products [38]. Breeders have long been interested in the metabolism of chlorophylls because of their crucial role in plant growth and development and human health [2,42]. Similar to previous findings [9,43], the *AcLhcb3.1/3.2* genes regulated chlorophyll content under biotic and abiotic stress. In tomatoes, researchers have reported that all the *suffulta* mutants for chlorophyll were segregated in the recessive mendelian manner in a reciprocal backcross with wild type plants [44]. Previously, researchers have incorporated genome-wide association study (GWAS) for connecting the desired trait to its underlying genetics [33,45]. It will be worthwhile to conduct GWAS for chlorophyll traits to improve our understanding of chlorophyll metabolism in plants [46].

## 4. Materials and Methods and Analysis

### 4.1. Genome-Wide Identification of LHC Genes in Kiwifruit

We retrieved whole genome sequences, coding sequences, and protein sequences for both kiwifruit species (Ac and Ae) from Kiwifruit Genome Database (http://kiwifruitgenome.org/ (accessed on 1 August 2021)). The protein sequences for *AtLHCs* were collected from the TAIR database (https://www.arabidopsis.org/ (accessed on 1 August 2021)). The local BLAST tool was used to construct the protein database of both kiwifruit species. The protein sequences of Arabidopsis LHC protein were used to query the kiwifruit protein database by the BLASTp. The candidates *AcLHC* and *AeLHC* were identified using a cutoff score of ≥100 and an e-value of ≤1 × e^−10^ for BLASTp. The Conserved Domain for kiwifruit LHCs was determined by utilizing the Conserved Domain Database (CDD) (https://www.ncbi.nlm.nih.gov/Structure/cdd/cdd.shtml (accessed on 3 August 2021)) and the simple modular architecture research tool (SMART) (http://smart.embl.de/ (accessed on 3 August 2021)). The protein sequences containing the Chloroa_b-bind domain were used in subsequent analysis.

### 4.2. Analysis of Kiwifruit LHC Protein Structure 

The length of the protein, theoretical isoelectric point (pI), grand average of hydropathicity (GRAVY), and molecular weight (MW) of the kiwifruit LHC proteins in the kiwifruit species were computed by the ExPASy server (http://web.expasy.org/protparam/ (accessed on 5 August 2021)).

### 4.3. Gene Structure, Motif Features, and cis-Elements Analysis

The genome sequences and coding sequences of the kiwifruit *LHC* genes were used to investigate gene structures using the Gene Structure Display Server (GSDS 2.0, http://gsds.cbi.pku.edu.cn/ (accessed on 8 August 2021)). A maximum of 12 conserved motifs for kiwifruit LHC proteins were identified using MEME (http://meme-suite.org/tools/meme (accessed on 8 August 2021)) [47]. The cis-regulatory in 1500 bp upstream sequence of kiwifruit *LHC* genes were predicted with the PlantCARE database [48].

### 4.4. Phylogenetic Analysis of LHCs 

The multiple sequence alignments of AtLHC, AcLHC, and AeLHC were performed using ClustalX with default parameters [49]. The phylogenetic tree was constructed by MEGA software (v7.0.26, download from https://www.megasoftware.net/ (accessed on 9 August 2021)) using the neighbor-joining (NJ) method with 1000 bootstrap replicates.

### 4.5. Chromosomal Location, Gene Duplication, and Synteny Analysis 

The location of kiwifruit *LHC* genes was extracted from the corresponding GFF file using an in-house Perl script, and the genes on chromosomes were visualized using MapGene2 Chrome (http://mg2c.iask.in/mg2c_v2.0/ (accessed on 9 August 2021)). The MCScan software was employed to identify duplication patterns of kiwifruit *LHCs* using default parameters [50]. The synonymous (Ks) and nonsynonymous (Ka) mutation rates of the duplicated *LHC* gene pairs were calculated using TBtools software [51]. The syntenic blocks for kiwifruit *LHCs* were produced using the MCScanX software with default parameters [50], and gene pairs of kiwifruit *LHCs* were visualized by TBtools [51].

### 4.6. Expression Analysis of Kiwifruit LHCs 

To investigate the expression profiles of kiwifruit *LHCs*, we collected four published RNA-seq data from NCBI (https://www.ncbi.nlm.nih.gov/ (accessed on 10 August 2021)) including samples from leaves, roots, different developmental stages of fruits, fruits treated with or without low temperature, leaves infected with pathogens (PRJNA691387, PRJNA277383, PRJNA514344, and PRJNA514180). All transcriptome datasets were re-analyzed using *Actinidia chinensis*, ‘Red5’ and *A. eriantha* ‘white’ cultivars as reference genomes [38,52]. The reads were aligned using the HISAT2 v2.0.1 [38,52]. The reads alignment was performed using the HISAT2 v2.0.1 [53], and the transcripts were assembled and quantified using the STRINGTIE v2.1.5 [54].

### 4.7. Plant Materials and Treatments

The fresh leaf samples for A. *eriantha* ‘Maohua no.1’ and *A. chinensis* ‘Hongyang’ ‘Donghong’ were collected from plants at the Germplasm Resources Nursery of Lushan Botanical Garden, Nanchang County, Jiangxi Province, China. A part of the samples was used for gene cloning, and the rest of the samples were used for callus induction [55]. The calluses were placed under light and dark conditions for subsequent study. The tobacco plants were grown under a 16/8-h-light/dark photoperiod, 250 μmmol photons m^−2^ s^−1^, 26 °C, and 55% humidity.

### 4.8. RNA Extraction and cDNA Synthesis

Total RNA was extracted with a Hipure Plant RNA Mini Kit (Magen, Shanghai, China), and cDNA was synthesized with a Trans Script One-Step gDNA Removal and cDNA Synthesis Super Mit (Transgen, Beijing, China) by following the manufacturer’s instructions.

### 4.9. Quantitative Real-Time PCR (qRT-PCR) Analysis

For RT-qPCR analysis, specific primers (Table S2) were designed based on the predicted sequences of the genes. The cDNA from kiwifruit leaves, callus, and fruit was used as a template to perform qRT-PCR. According to the manufacturer’s instructions, the reaction mixture was prepared using Perfect Start Green qPCR SuperMix (Transgen, Beijing, China). Each sample was replicated thrice to minimize inherent errors. Additionally, each biological replicate contained three technical replicates. The actin gene was used as an internal control to calculate the ΔCt values of target genes. The relative expression was calculated by the 2^−^^ΔΔCt^ method and the specificity of the amplification was determined from the melting curve [56].

### 4.10. Subcellular Localization of AcLHCs and Transformation

The coding sequences (CDS) of the *AcLHCs* without stop codon were ligated into the pGreen vector to construct an AcLHC-eGFP fusion protein expression vector. In contrast, the empty pGreen vector was used as a negative control. For subcellular localization, constructed vector was introduced into *A. thaliana* plant by *Agrobacterium tumefaciens* (strain EHA105) [12]. Transformation assays in cell suspension culture of *Arabidopsis thaliana* leaves were performed under a laser scanning confocal microscope (Olympus IX83, Olympus company, Beijing, China) as described previously [57,58].

### 4.11. Transient Over-Expression Analysis in Tobacco Leaf 

For transient overexpression, we followed the same experimental procedure used for subcellular localization to construct the vectors. The experiment was performed in 6-weeks-old plants by selecting leaves at the fourth internode (counting from the shoot tip) for bacterial culture introduction. Chlorophyll contents and SPAD value were determined in transformed leaves according to previously described methods [59].

### 4.12. Statistical Analysis

All data were analyzed with GraphPad Prism 9 software (v9.0.0.121, purchased from https://www.graphpad.com/scientific-software/prism/ (accessed on 9 August 2021)). One-way ANOVA and two-way ANOVA were performed to check the significance level of the data. Tukey’s tests were used to compare the mean differences [60]. The mean differences were considered statistically significant at *p* < 0.05.

## 5. Conclusions

The *LHC* superfamily members are reported to participate in the plant photosynthesis process. The present study carried out the evolutionary gene relationships, protein motifs, structure, and expression profiles to characterize *LHC* superfamily members in Ac and Ae genomes. The subcellular localization analysis revealed that *LHC* genes were located in the chloroplast. Quantitative RT–qPCR analysis showed that *LHC* genes were preferentially expressed in the leaf. Additionally, the functional validation results showed that AcLhcb3.1/3.2 improved chlorophyll a content in tobacco leaves. Overall, our findings provided valuable insights into evolutionary patterns and functional diversity of *LHC* superfamily members and provided candidate genes for future breeding programs of kiwifruit. Predicting highly correlated loci for chlorophyll content in plant genomes through artificial intelligence techniques coupled with multi-omics studies can open new avenues for researchers to improve the understanding of chlorophyll metabolism in plants [61,62].

## Supplementary Materials

The following supporting information can be downloaded at: https://www.mdpi.com/article/10.3390/ijms23126528/s1.
