# Supplementary figures and images for: Genome-Wide Identification of the LHC Gene Family in Kiwifruit and Regulatory Role of AcLhcb3.1/3.2 for Chlorophyll a Content
Source: Int J Mol Sci. 2022 Jun 10;23(12):6528. doi: 10.3390/ijms23126528 (PMC9224368; doi:10.3390/ijms23126528)

(A)

Ac

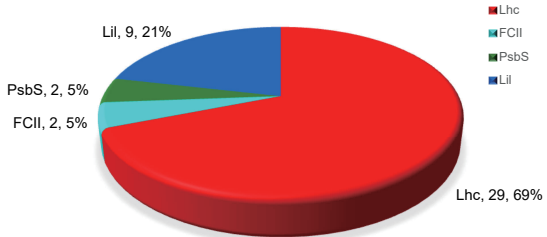

(B)

Ae

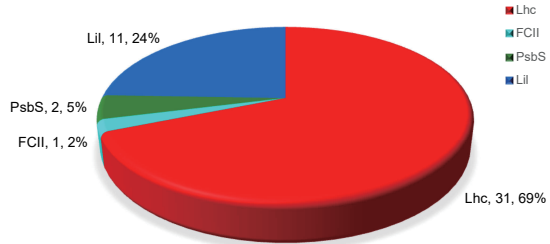

Supplement: Supplementary file 1 [file ijms-23-06528-s001.zip › Figure S1.pdf]

(A)

Ac

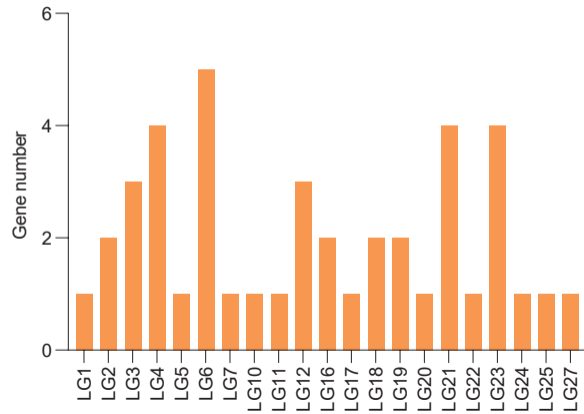

(B)

Ae

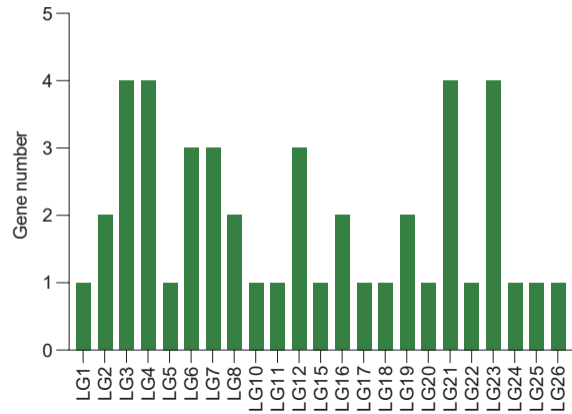

Supplement: Supplementary file 1 [file ijms-23-06528-s001.zip › Figure S2.pdf]

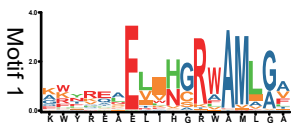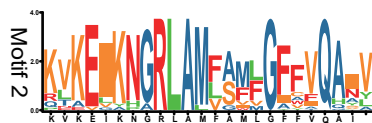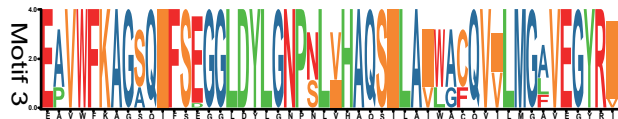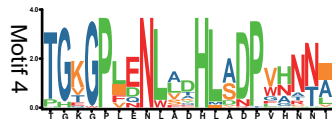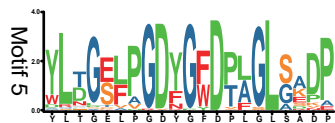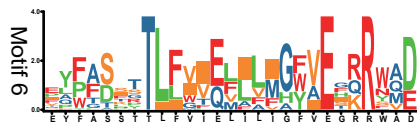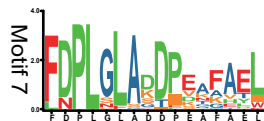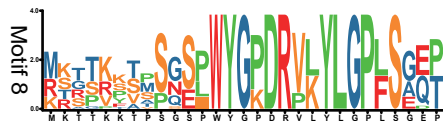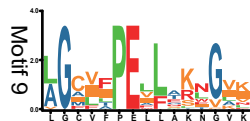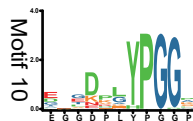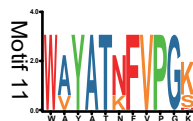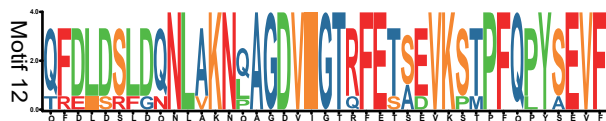

Supplement: Supplementary file 1 [file ijms-23-06528-s001.zip › Figure S3.pdf]

(A)

Ac

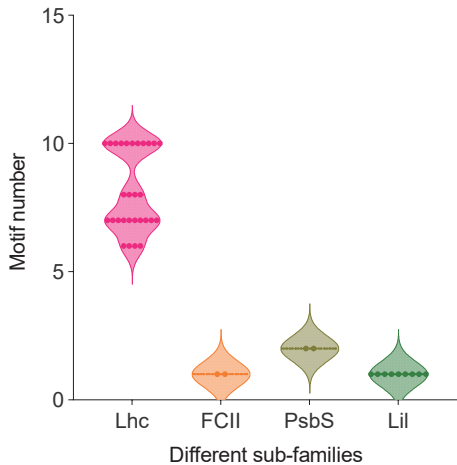

(B)

Ae

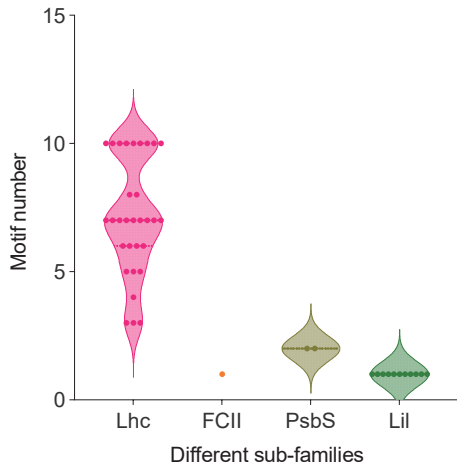

Supplement: Supplementary file 1 [file ijms-23-06528-s001.zip › Figure S4.pdf]

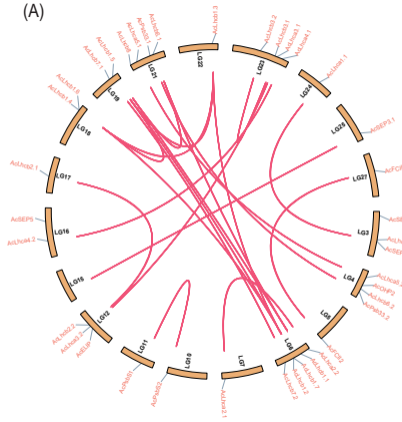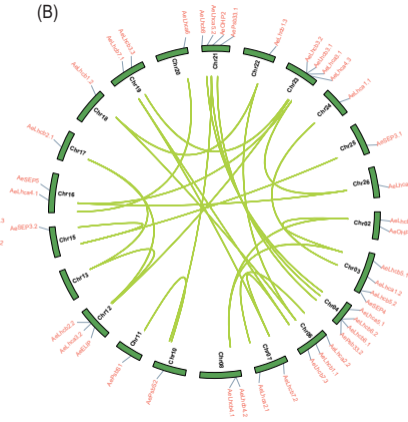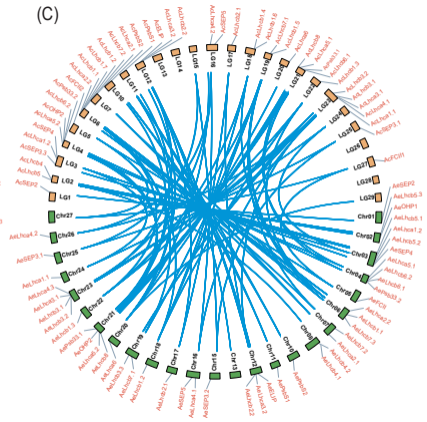

Supplement: Supplementary file 1 [file ijms-23-06528-s001.zip › Figure S5.pdf]

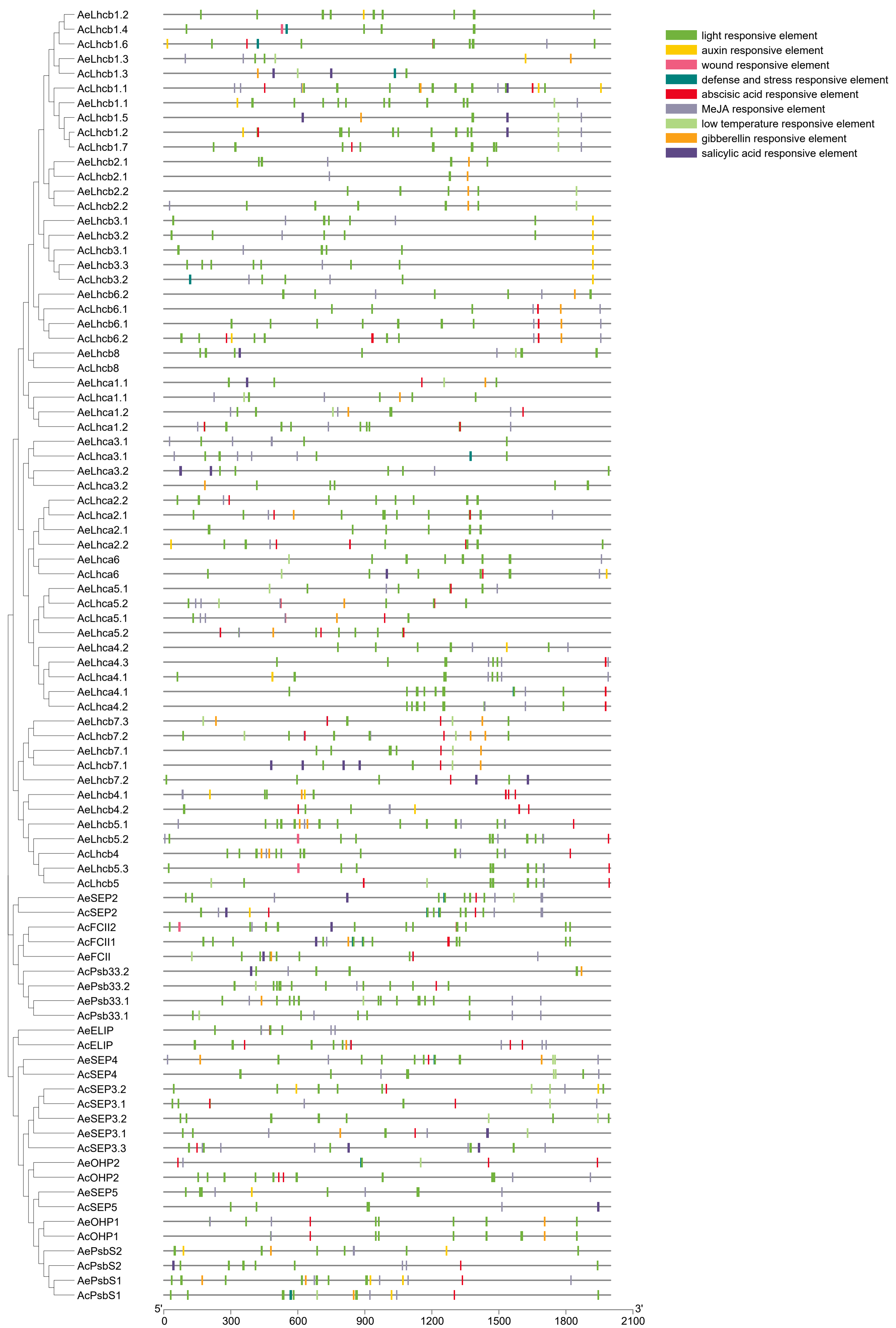

Supplement: Supplementary file 1 [file ijms-23-06528-s001.zip › Figure S6.pdf]

DY

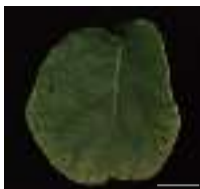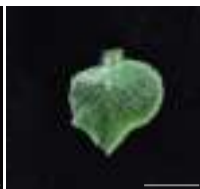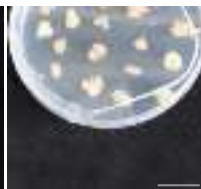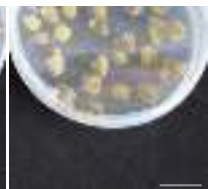

HY

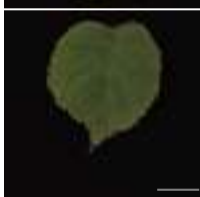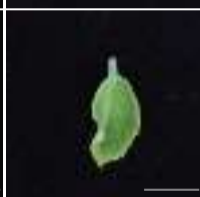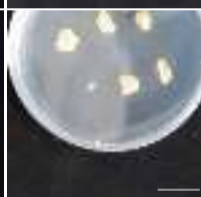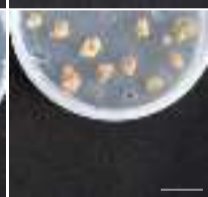

MH

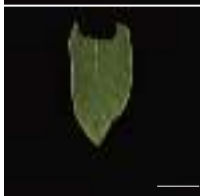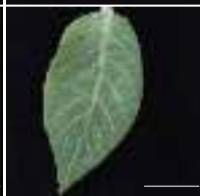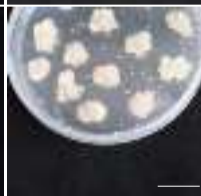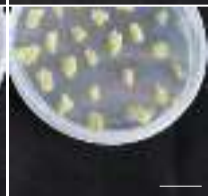

OL

YL

CD

CL

Supplement: Supplementary file 1 [file ijms-23-06528-s001.zip › Figure S7.pdf]

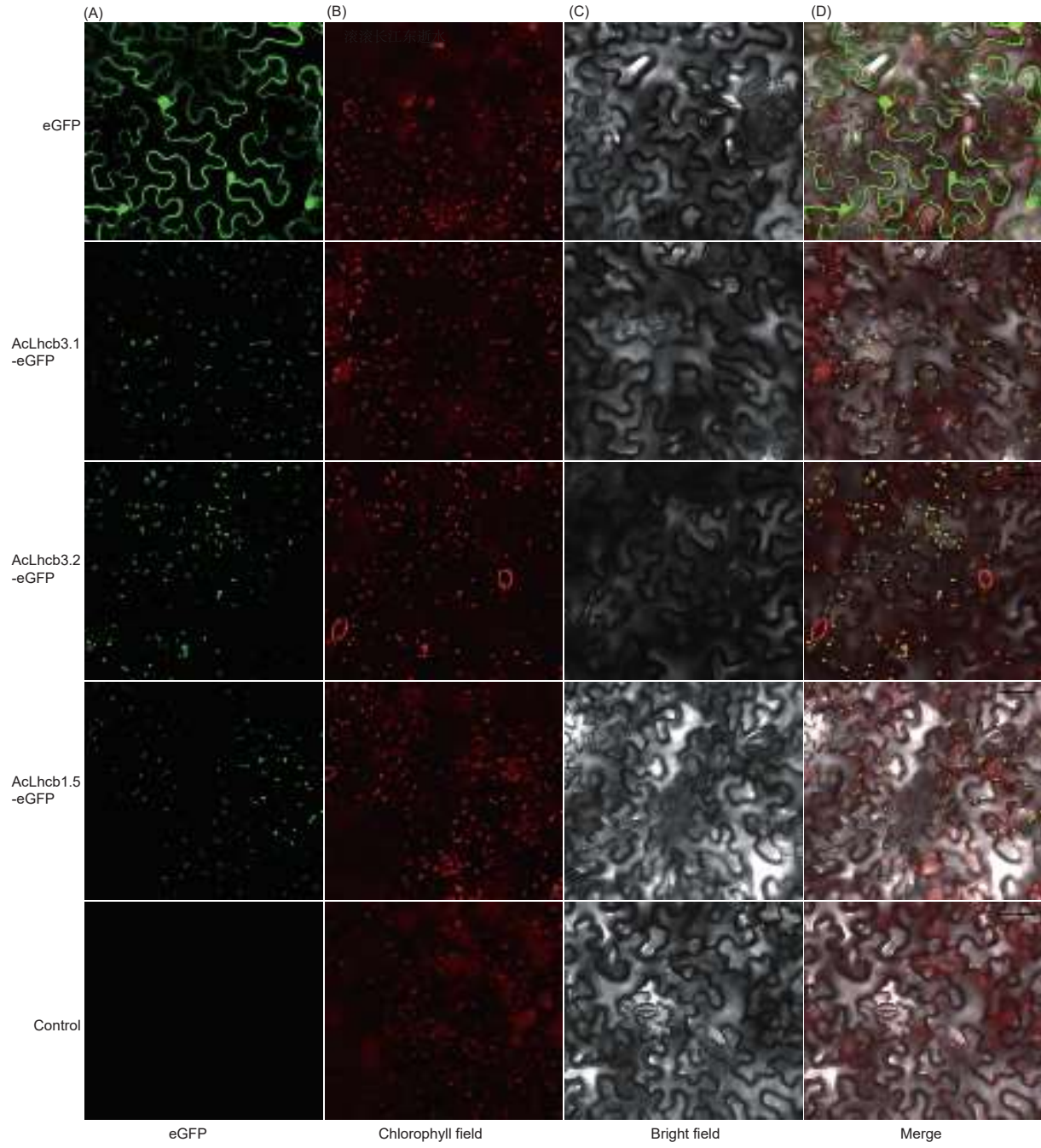

Supplement: Supplementary file 1 [file ijms-23-06528-s001.zip › Figure S8.pdf]

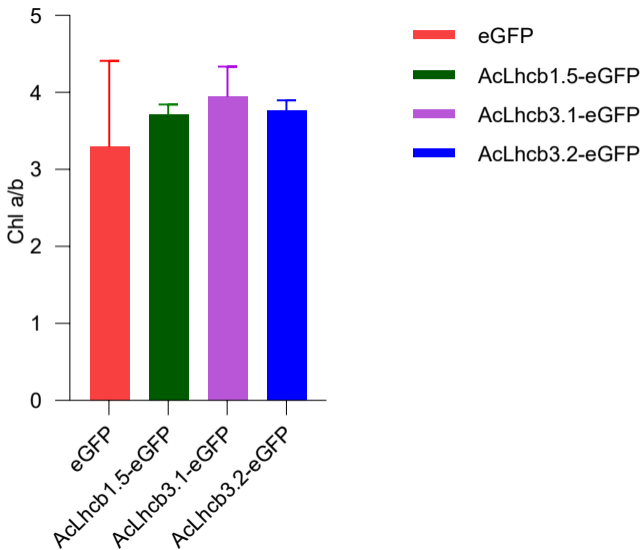

Supplement: Supplementary file 1 [file ijms-23-06528-s001.zip › Figure S9.pdf]
